# The relation between epistemic trust and borderline pathology in an adolescent inpatient sample

**DOI:** 10.1186/s40479-019-0110-7

**Published:** 2019-08-28

**Authors:** William Orme, Lauren Bowersox, Salome Vanwoerden, Peter Fonagy, Carla Sharp

**Affiliations:** 1 Baylor College of Medicine, The Menninger Clinic, Houston, Texas USA; 2 University of Houston, Houston, Texas USA; 3 Research Department of Clinical, Educational and Health Psychology, University College London, London, UK

**Keywords:** Epistemic trust, Borderline personality disorder, Mentalization

## Abstract

**Background:**

Recent extensions of mentalization theory have included the hypothesis that a reduced capacity for epistemic trust in the context of attachment relationships may represent a core vulnerability for the development of borderline personality disorder (BPD). The first aim of the current study was to explore empirical relationships between epistemic trust and symptoms of BPD. The second aim was to explore the effect of epistemic trust on treatment response.

**Methods:**

Data were collected from 322 inpatient adolescents. The Inventory of Parent and Peer Attachment (IPPA) trust in mother and father subscales were used to approximate epistemic trust in the absence of a validated measure. A multimodal approach was used to measure BPD including self-report, parent-report, and interviewer ratings. Regression analyses were performed to explore the relationship between IPPA trust scores and measures of BPD. Mixed-design analyses of variance were conducted to evaluate whether self-reported parent trust at admission influenced progress in treatment.

**Results:**

As hypothesized, results indicated that reduced IPPA trust in parents correlated with BPD symptoms across various measures. Levels of IPPA trust in parents at admission did not moderate a reduction in BPD symptoms over the course of treatment.

**Conclusions:**

This study provides support for the theoretical association between deficits in epistemic trust and BPD while also highlighting the need for a validated measure of epistemic trust. Although parent trust at admission did not moderate a reduction in BPD symptoms over the course of treatment, this result may suggest that progress in treatment, and perhaps the ability to cultivate trust in the treatment setting and providers, may not be overly determined by levels of parent trust.

## Background

Borderline personality disorder (BPD) is a severe condition that is often associated with life-long suffering [1]. Based on evidence from a factor analysis of personality disorder symptoms, Sharp et al. [2] suggested that BPD symptomology may be representative of common or shared features of all personality pathology, highlighting the central importance and therapeutic utility of researching and treating BPD. Evidence suggests that symptoms of BPD may be as clinically relevant during the adolescent years as in adulthood [3, 4], which has led researchers to define BPD as a lifespan disorder [5, 6]. A number of treatments have demonstrated clinical utility for adolescents with BPD, such as cognitive analytic therapy [7, 8], mentalization-based treatment (MBT) [9, 10], dialectical behavioral therapy (DBT) [11, 12], transference-focused psychotherapy (TFP) [13, 14], and early intervention programs [8, 15]. The potential severity of BPD in adolescence and the clinical interest in offering viable treatments highlight the need to identify correlates of BPD that may serve as early intervention targets [4, 16].

Epistemic trust has been proposed to be a key treatment target [4, 17–19] Epistemic trust is defined as the ability to appraise incoming information from the social world as accurate, reliable, and personally relevant, allowing for the information to be incorporated into existing knowledge domains [20, 21]. Epistemic trust has been discussed in conjunction with epistemic vigilance, which Sperber et al. [21] described as a natural human capacity derived from the evolutionary necessity of guarding against misinformation so that reliable, culturally-transmitted knowledge may be acquired and used to maintain a competitive edge in the world. They suggested that vigilance and trust are calibrated depending on the situation, communicator, and information, with the underlying belief that humans are more vigilant than inherently trusting. Fonagy et al. [20] asserted that one of the primary ways that epistemic vigilance is overcome is through sensitive caregiving in the context of secure attachment relationships. In relationships such as these, parents consistently adopt a mentalizing stance toward the child by seeing the child as an intentional individual and attempting to make sense of the child’s behavior as arising from underlying mental states [22, 23]. The parent conveys understanding of the child’s subjective experience in a way that is accurate (i.e., personally relevant) and explicitly marked as the parent’s representation of the child’s mental state [23]. Marked communication, through appropriate eye contact, turn-taking, and intonation, may serve as an *ostensive cue* [24] that signals to the child that socially generalizable and personally relevant information will be communicated, effectively inviting the child to pay attention and suspend epistemic vigilance to make use of new social information [18, 20]. In the absence of marked communication, epistemic vigilance may persist or even increase when abuse or neglect is involved [20], although no studies have directly tested this hypothesis to date. However, this hypothesis is consistent with research and theory demonstrating that childhood trauma is associated with significant impairments in mentalizing [23, 25, 26]. If epistemic trust increases, through the use of attuned communication, it is expected to benefit the individual when the communication occurs within a benign social context, where knowledge is accurately and honestly represented allowing for the transfer of helpful and relevant information [19]. However, if the increase in epistemic trust occurs within a harmful context where information is distorted, then gains in epistemic trust would come at an overall cost to psychological functioning. Fonagy et al. [20] suggested that chronic epistemic mistrust may contribute to the rigidity that is common in personality pathology in general and BPD in particular. When individuals do not trust in the reliability or relevancy of interpersonal communication, their mistrust may lead to inflexible adherence to existing beliefs, perspectives, or behaviors.

Theoretically, epistemic trust may represent a compelling treatment target but few studies have empirically examined the construct in the context of personality pathology. Some evidence has been found that may challenge the theory, suggesting that individuals automatically accept new information before determining its veracity and usefulness [27, 28]. However, other studies have found evidence of epistemic vigilance especially when information is personally relevant [29]. One of the few studies to specifically explore epistemic trust related to attachment [30] found compelling evidence for epistemic vigilance in young children, varying based on their attachment classification. Specifically, when children heard conflicting claims from their mothers and strangers in a series of tasks, securely attached children tended to trust information from their mothers when the claims were reasonable, while also demonstrating the ability to trust their own perception when claims were less reasonable. Conversely, insecurely attached children showed issues with epistemic trust with the most pronounced deficits emerging in children classified as insecure-disorganized, who demonstrated suspicion of the claims of both their mothers and strangers. Regarding epistemic trust and BPD, there have been no known empirical studies to date, but there has been research on trust, more generally defined. For example, individuals with BPD have been found to appraise neutral or non-emotional faces as untrustworthy, which is partially mediated by how sensitive individuals with BPD are to rejection [31]. Numerous studies have employed economic trust games as a way to better understand how individuals with BPD make decisions related to trust and risk taking. For instance, individuals with BPD have been found to be less trusting or less likely to invest in mutually beneficial relationships during a trust game [32]. Liebke et al. [33] found that when individuals with BPD were given explicit indicators of social acceptance in a pre-game virtual encounter, they demonstrated reduced cooperation in a trust game and did not revise their existing low expectations of social acceptance, which suggests an inherent distrust in positive social feedback. Problems in cooperation, perception of fairness, trust, and repairing of interpersonal ruptures have been found to be associated with abnormal activation in the anterior insula, cingulated cortex, and amygdala [34, 35]. These studies suggest that while research has yet to be conducted on epistemic trust explicitly, existing evidence points to a robust relationship between mistrust and borderline pathology in adults. This research has yet to be extended to adolescent borderline pathology despite the fact that BPD typically onsets in adolescence [4].

Understanding the role of trust during adolescence is important given the unique developmental changes occurring during this time, especially in executive functioning, perspective taking, emotion regulation, risk-taking, and identity [36, 37]. Trust may be particularly sensitive during this time, susceptible to further developmental setbacks or to meaningful growth. Research has shown that younger adolescents demonstrate less trust and reciprocity than older adolescents [38]. Evidence suggests that gains in trust with age may be due to improved emotion regulation capacities, especially the regulation of anger, allowing for more resilience in the face of violations of trust [39]. Yet, individual differences in emotion regulation already present in childhood may be magnified during adolescence [40], leaving the development of trust during these years vulnerable to disruption. Changes in certain brain regions that facilitate perspective taking and increased reciprocity may also impact the development of trust during adolescence [41]. Given the significant changes in neurodevelopment and social cognition during adolescence, research into conditions of impaired self-other relatedness, such as BPD, may allow factors to be identified that contribute to improved functioning.

Against this background, the first aim of this study was to examine the relationship between epistemic trust and borderline pathology in an adolescent inpatient sample. We hypothesized that epistemic trust would be negatively associated with borderline pathology after controlling for known covariates of BPD. In the absence of a validated measure for epistemic trust, the *Inventory of Parent and Peer Attachment* (IPPA) [42] trust in mother and father scales were used. Consistent with theorization about attachment facilitating epistemic trust [18, 20], the IPPA trust scales were formulated from within an attachment framework and measure the degree to which adolescents experience their mothers and fathers as understanding, accepting, and responsive. Trust as operationalized by the IPPA scales captures elements that are thought to be facilitative of epistemic trust, such as the expectation of being understood. This conceptualization is different than epistemic trust, which is specifically focused on the ability to appraise social communication as reliable, useful, and personally relevant. However, given that measures of epistemic trust have yet to be developed, the IPPA was judged to be suitable to provide data with close enough relevance to the topic for preliminary analyses. The second aim of the study was to examine the impact of epistemic trust on responsiveness to treatment. Fonagy et al. [20] suggested that within a benign social context individuals with high epistemic trust may make better use of new social information and demonstrate greater flexibility than those with low epistemic trust. Consequently, levels of epistemic trust may impact the extent to which individuals utilize, and benefit from, treatment resources. We hypothesized that within an inpatient context, individuals with fewer trust deficits in their parents (i.e., higher baseline trust as measured by the IPPA) would be more likely to make treatment progress based on the assumption that they may be able to cultivate trust in the treatment setting and providers with greater ease.

## Methods

### Participants

The sample consisted of adolescents who were consecutively admitted to a private psychiatric hospital that serves individuals with severe behavioral and emotional disorders. Inclusion criterion was sufficient proficiency in English to consent and complete assessments, and exclusion criteria were a diagnosis of a psychotic disorder, an autism spectrum disorder, or an IQ of less than 70. Of *N* = 567 adolescents and their families who were approached for consent, *n* = 41 declined and *n* = 65 were excluded based on aforementioned criteria. Additionally, *n* = 139 were missing data on main study variables and were therefore excluded from analyses. Therefore, the final sample consisted of *N* = 322 adolescents ranging in age from 12 to 17 years old (*M* = 15.33; *SD* = 1.398). The gender composition of the sample was 67.4% female (*n* = 217) and 32.6% male (*n* = 105). The sample identified as 86.1% Caucasian, 3.4% Asian, 2.4% African-American, and 8.1% Multiracial or other. The sample was generally drawn from a high socioeconomic background, with over 50% of the sample reporting a household income of $150,000 or more. The average length of stay on the inpatient unit was 36.5 days (*SD* = 13.6). In regard to psychopathology, 37.9% (*n* = 122) qualified for diagnosis of borderline personality disorder. Other forms of psychopathology were also prevalent with 66.3% (*n* = 205) of respondents qualifying for a mood disorder (i.e., major depression, dysthymia, hypomania, or mania) and 60.2% (*n* = 194) meeting criteria for an anxiety (GAD, phobias, panic), OCD, or trauma disorder.

### Measures

#### Inventory of parent and peer attachment (IPPA) [42]

The IPPA mother trust (IPPA-M) and father trust (IPPA-F) scales were selected to approximate epistemic trust. The IPPA is a 75-item self-report measure developed to assess the perceived quality of attachment relationships with mother, father, and peers. The IPPA is evaluated on a 5-point Likert scale (1 = *almost never or never true*; 5 = *almost always or always true*) and has three subscales (trust, communication, and alienation) that target different factors impacting the quality of attachment relationships. The ten items of the trust scale measure various dimensions of general trust. Four items, “My mother understands me,” “When we discuss things, my mother cares about my point of view,” “When I am angry about something, my mother tries to be understanding,” and “My mother respects my feelings,” capture the anticipation of attuned, contingent, curious, and understanding communication. Three items, “My mother accepts me as I am,” “My mother trusts my judgment,” and “My mother expects too much from me,” (reverse scored) reflect an expectation of nonjudgment, mutuality, and fairness. The final three items, “I feel my mother does a good job as my mother,” “I wish I had a different mother,” (reverse scored) and “I trust my mother,” address a broader sense of parent reliability. The IPPA trust construct is broader than conceptualizations of epistemic trust, which are focused more specifically on trust in the reliability of communicated knowledge. However, it was assumed that individuals with epistemic trust deficits would likely respond to IPPA items in a similar manner, providing data that would be germane to theoretical conceptualizations in the absence of an epistemic trust measure. In the current sample, the internal consistency for both IPPA-M (*α* = .94) and IPPA-F (*α* = .94) were high.

#### Borderline personality features scale for children, child Report (BPFS-C) [43]

The BPFS-C is a self-report questionnaire assessing BPD features for youth ages 9–18. The BPFS-C was adapted from the BPD scale of the Personality Assessment Inventory [44] for use in youth. The BPFS-C contains 24 items, which are rated on a 5-point Likert scale (1 = *not true at all*; 5 = *always true*). Sample items include “I want to let some people know how much they’ve hurt me,” and “When I’m mad, I can’t control what I do.” The BPFS-C has demonstrated evidence for criterion and concurrent validity [45, 46]. In the current sample, internal consistency was good (α = .89).

#### Borderline personality features scale for children, parent report (BPSF-P) [47]

The BPFS-P was adapted from the BPFS-C for parent reports. The BPFS-P directly mirrors the child-reported version in item content and scale. The BPFS-P has demonstrated evidence for criterion and concurrent validity among adolescents [47]. In the current sample, the BPFS-P demonstrated good internal consistency (α = .88).

#### Child interview for DSM-IV borderline personality disorder (CIBPD) [48]

The CIBPD is a semi-structured diagnostic interview for DSM-IV BPD developed for use with children and adolescents. The interview covers the nine DSM-IV criteria with corresponding prompts used by the interviewer to investigate that criterion, which are then rated with a score of 0 (absent), 1 (probably present), or 2 (definitely present). Adolescents meeting at least five criteria at the 2-level meet diagnostic criteria for a CIBPD-defined categorical diagnosis of BPD. For the current study, we utilized both the categorical diagnosis of BPD as well as the total score as a dimensional measure of BPD features, which is a sum of scores for each of the 9 criteria (maximum score of 18). Excellent psychometric properties of this measure including interrater reliability and concurrent validity have been demonstrated in adolescents [49]. Internal consistency in the current sample was adequate (α = .77).

#### The child behavior checklist (CBCL) [50]

The CBCL is a well-established broad-band questionnaire of psychopathology completed by parents of adolescents. The measure contains 112 problem items, each scored on a 3-point Likert scale (0 = *not true*, 2 = *very or often true*). The measure yields a number of scales, some of which are empirically derived and some theoretically based, as well as three higher order factors: Total Problems, Internalizing, and Externalizing. All scales were converted to T-scores. In the current study, the Total Problems scale was used as an index of overall psychiatric severity. Internal consistency in the current sample was excellent (α = .94).

### Procedures

The study was approved by a human subjects review committee, and subjects participated after signing a written voluntary informed consent form. Adolescents were collectively assessed by doctoral-level clinical psychology students and/or trained clinical research assistants. Assessments were conducted independently and in private within the first 2 weeks following admission.

### Data analytic strategy

The first aim of the study was to explore the relationship between epistemic trust, as approximated by the IPPA trust scales, and borderline symptoms. This was accomplished by first calculating the zero-order correlations between IPPA scores and all measures of borderline pathology. We included age, gender, and general psychopathology in the correlation matrix in light of their known associations with borderline symptoms [49, 51–54]. Next, we ran a series of regression analyses. Linear regressions were used for all continuous dependent variables while a binary logistic regression was used for the single categorical dependent variable (i.e., CIBPD). The second aim of the study was to evaluate whether baseline levels of trust in parents would impact the course of treatment. We ran two separate mixed design analyses of variance to evaluate whether IPPA trust scores moderated a reduction in BPD symptoms from admission to discharge. Because the BPFS-C was the only measure of BPD symptoms administered at both admission and discharge, it was utilized as the dependent variable. In this design, we evaluated within-person effects of change in BPD symptoms over the course of treatment as well as the between-person effect of trust with either mothers or fathers in separate models. Interaction effects between IPPA trust scores and change in BPD symptoms from admission to discharge were evaluated.

## Results

### Attrition analyses

The final sample (*N* = 322) was compared against those who were excluded for not completing the IPPA (*n* = 139) to assess for possible group differences. No significant differences were found in age, gender, general psychopathology, or measures of BPD except for the categorical CIBPD measure. Those who completed the IPPA mother trust scale and IPPA father trust scale had significantly more individuals qualifying for a diagnosis of BPD than those who did not complete the measures (*p* = .034 and *p* = .016, respectively).

### Bivariate relations between study variables

All variables were found to be normally distributed in initial data screenings. No univariate or multivariate outliers were detected. Bivariate correlations were conducted (Table 1) among variables of interest. Correlations showed significant inverse relationships between mother and father trust and all measures of BPD, with the exception of mother trust and BPFS-P which was uncorrelated. Although age was unrelated to trust and BPD measures, gender was strongly correlated with all measures of BPD, with females associated with higher levels of BPD symptoms. General psychopathology, as measured by the total score of the CBCL, showed a positive relationship with all measures of BPD and an inverse relationship with age. Gender was also correlated with total CBCL scores, with females associated with higher levels of general psychopathology.

**Table 1:** Bivariate correlations among variables

Variable	1	2	3	4	5	6	7	8	9
1. Age	---								
2. Gender	.11**	---							
3. IPPA-M	−.01	.03	---						
4. IPPA-F	.01	.00	.38***	---					
5. BPFS-C	−.07	−.18***	−.18***	−.13*	---				
6. BPFS-P	−.06	−.14***	−.09	−.15**	.22***	---			
7. CIBPD (categorical)	−.04	−.25***	−.22***	−.16**	.46***	.28***	---		
8. CIBPD (dimensional)	−.05	−.26***	−.20***	−.12*	.60***	.31***	.81***	---	
9. CBCL	−.10*	−.08*	.03	−.09	.17***	.69***	.17***	.22***	---

*IPPA-M* Inventory of Parent and Peer Attachment, Mother Trust Subscale, *IPPA-P* Inventory of Parent and Peer Attachment, Father Trust Subscale, *BPFS-C* Borderline Personality Features Scale, Child Report, *BPFS-P* Borderline Personality Features Scale, Parent Report, *CIBPD* Child Interview for DSM-IV Borderline Personality Disorder, *CBCL* Child Behavior Checklist

*p < .05; **p < .01; ***p < .001

### Relation between IPPA trust and borderline pathology controlling for age, gender, and other psychopathology

To explore the relationship between IPPA trust and BPD beyond the bivariate level, a series of regression analyses were conducted. Regression assumptions were checked and verified; no issues were found with multicollinearity, heteroscedasticity, or linearity. Table 2 displays the results from linear regressions conducted on dimensional dependent variables and the binary logistic regression conducted on the categorical measurement of BPD using the CIBPD. In support of the research hypotheses, the results revealed that even after controlling for age, gender, and general psychopathology, self-reported mother trust negatively correlated with levels of BPD symptoms and with a categorical BPD diagnosis as defined by the CIBPD. Similarly, father trust was negatively associated with borderline symptoms as measured by all the dependent variables except for the dimensional CIPBD score, which approached significance (*p* = .053). Father trust was also negatively associated with a categorical diagnosis of BPD as defined by the CIBPD.

**Table 2:** Regression beta weights

Variable	BPFS-C	BPFS-P	CIBPD (dimensional)	CIBPD (categorical) ^a^
IPPA-M	−.19***	−.11*	−.21***	−.05***
Age	−.10	−.05	−.08	−.13
Gender	−.08	−.04	−.19**	1.00***
CBCL	.06	.67***	.15**	.06**
IPPA-F	−.12*	−.10*	−.11	−.03*
Age	−.08	−.04	−.06	−.11
Gender	−.10	−.04	−.20***	.97***
CBCL	.04	−.10*	.14*	.02*

*IPPA-M* Inventory of Parent and Peer Attachment, Mother Trust Subscale, *IPPA-P* Inventory of Parent and Peer Attachment, Father Trust Subscale, *BPFS-C* Borderline Personality Features Scale, Child Report, *BPFS-P* Borderline Personality Features Scale, Parent Report, *CIBPD* Child Interview for DSM-IV Borderline Personality Disorder, *CBCL* Childhood Behavioral Checklist

^a^Binary logistic regression beta weights

*p < .05; **p < .01; ***p < .001

### Evaluating IPPA trust as moderator of the reduction in borderline pathology from admission to discharge

To evaluate the second aim, two separate mixed design ANOVAs were run. The first analysis examined change in BPFS-C scores from admission to discharge as the within-subjects factor moderated by level of trust in mothers at admission. There was a significant main effect of time, *F* (1, 249) = 30.77, *p* < .001. Examination of descriptive statistics revealed that individuals’ decreased in their BPD symptoms from admission to discharge. The interaction effect between time and trust in mothers on BPFS-C scores was insignificant, *F* (1, 249) = 8.52, *p* = .73. This indicates that level of trust in mothers at admission did not have an effect on reduction in BPD symptoms over the course of treatment. The same mixed design ANOVA was run with trust in fathers at admission included as the between-subjects factor. Again, the main effect of time was significant, *F* (1, 239) = 30.43, *p* < .001; however, the interaction between time and trust in fathers on BPFS-C scores was again insignificant, *F* (1, 239) = 1.28, *p* = .26. Therefore, it was concluded that trust in fathers at admission did not have an effect on reduction in BPD symptoms from admission to discharge in this sample.

## Discussion

A primary aim of this study was to examine the relationship between epistemic trust and borderline pathology in a sample of adolescent inpatients while controlling for known covariates of BPD. Given the absence of a validated measure of epistemic trust to date, the construct was operationalized using IPPA trust scales. Therefore, the results pertain to trust more broadly defined within an attachment framework, capturing the anticipation of parents as being understanding, reasonable, respectful, and reliable. BPD symptoms were measured using a multi-method approach, including self-report, parent-report, and interviewer ratings. BPD scores were calculated both categorically, representing whether participants qualified for a full diagnosis of BPD according to the CIBPD criteria, as well as dimensionally, indicating the degree to which they evidenced symptoms of BPD. After controlling for age, gender, and general psychopathology, adolescent trust in mothers was negatively associated with all BPD measures, and trust in fathers was negatively associated with all BPD scores, except one (i.e, the CIBPD score) that approached significance.

Although these findings pertain to trust in parents more generally defined, the findings coincide well with the theoretical connection between deficits in epistemic trust and vulnerability to borderline pathology. Although existing research on BPD has predominately focused on trust in the context of simulated social exchange games [32, 34, 35, 55] or through facial appraisal tasks [31], this study adds to existing research by linking deficits in adolescent expectation of understanding and perspective-taking from parents with the likelihood of BPD symptoms. These data support theoretical formulations that suggest individuals who anticipate misattuned or insensitive communication may maintain epistemic vigilance and inflexibly hold to existing perspectives or behaviors leading to the personality rigidity that is common in BPD [18, 20]. The results are also consistent with Sharp and Fonagy’s [4] suggestion that epistemic trust may represent an important early intervention target for BPD given the characteristic difficulty that individuals with this condition have in adjusting their viewpoints in response to new social information. A deficit in parental trust may be a strong signal, if not a potential source, of emergent borderline pathology.

Existing treatments paradigms for BPD may be augmented by incorporating a focus on epistemic trust. Fonagy and Allison [18] proposed that reconstituted epistemic trust, emerging within the context of sensitive mentalizing, may build the patient’s expectation of social learning and mollify previously entrenched vigilance. The renewed potential for social learning is key if patients are to benefit from the knowledge, skills, and resources that therapists have to offer. Perhaps more importantly, renewed epistemic trust may allow patients to benefit from social exchanges outside of therapy, unlocking a previously blocked conduit of information that is useful for their functioning and well-being. Fonagy and Allison argued that although all effective treatments for BPD likely derive benefits from improved mentalizing, the focus of treatment should not be on enhancing mentalizing. Rather, mentalizing is important insofar as it creates favorable conditions for the restoration of epistemic trust. Accordingly, therapists may benefit from anticipating that many individuals with BPD have a baseline vigilance that, unless centrally addressed, may stymie acquisition of skills and overall progress. Therapists are encouraged to devote explicit time and energy to understanding and reflecting the patient’s subjectivity using sensitive, marked, and mirrored communication [23] to develop epistemic trust. Therapists can be encouraged that thoughtful articulation of the patient’s subjectivity may be valuable in itself for the benefit of softening vigilance and opening capacities for social learning.

The second hypothesis was that higher levels of trust in parents upon admission would translate into better treatment gains based on the assumption that greater trust within a family context might extend to the treatment environment leading to enhanced utilization of interventions. Although there was a significant improvement in self-reported symptoms of BPD between admission and discharge, baseline levels of trust in parents did not moderate this relationship. This result highlights the efficacy of inpatient treatment for this population but did not support the initial hypothesis. Rather, the finding suggests that individuals with borderline pathology were able to make progress in treatment regardless of initial levels of trust in parents. One way to understand this outcome is that it may reflect the non-determinant nature of trust. Just as attachment and mentalization vary across context rather than representing fixed capacities [56–58], levels of trust likely vary per context as well, which is consistent with theory [18]. Variability in the capacity to trust may actually be a critical component to therapeutic progress [19]. That adolescents were able to make progress in treatment in this study, despite preexisting trust deficits, suggests treatment settings and providers have the ability to facilitate the emergence of trust that was limited in other contexts. In fact, it is the degree of change in epistemic trust, facilitated through treatment settings and providers, that is likely predictive of a reduction in BPD symptomology, rather than initial levels of parent trust [19]. To test this hypothesis, researchers should consider collecting pre- and post-discharge trust scores in order to evaluate the degree of change for more detailed analyses.

### Limitations

A key limitation of this study lies in the use of IPPA to operationalize epistemic trust. The IPPA trust scales were conceptualized within an attachment framework [42], which aligns well with the proposition that attachment relationships may be a primary context in which epistemic trust is fostered [20]. Although the IPPA trust scales capture dimensions of attachment relationships that may be necessary precursors to developing epistemic trust (e.g., perceived understanding and perspective-taking abilities), the scale only approximates epistemic trust, which has a more specific focus on the ability to appraise communication as authentic, reliable, and personally meaningful [20]. Although the results from this study are not inconsistent with theory of epistemic trust, a more accurate test of the theory cannot occur until a dedicated measure of epistemic trust is developed for use in future research. In the meantime, additional research exploring the relationship between trust and borderline pathology using other existing measures that may approximate epistemic trust (e.g., the children’s generalized trust belief scale [59]) may be helpful in continuing to build our knowledge base in this area.

In addition to the aforementioned points, other limitations of this study should be noted. First, analyses indicated that the final sample had a significantly higher percentage of individuals who qualified for a full diagnosis of BPD than those who were excluded. Although group differences were not found on other measures of BPD, this finding suggests that a bias could not be ruled out in results due to an underrepresentation of individuals who did not meet full criteria for BPD. Second, significant inverse relationships between parent trust and BPD symptoms were found in cross-sectional data only, preventing any causal links to be drawn. Third, the generalizability of the significant findings of this study is limited given that IPPA scores were drawn from adolescent inpatients upon admission to a psychiatric unit. It cannot be assumed that the relationship between parent mistrust and BPD symptoms holds true in other groups of individuals, such as adults or individuals in outpatient treatment. Similarly, the sample in this study was composed of predominately Caucasian individuals from affluent socioeconomic backgrounds. Future research into the nature of epistemic trust in diverse outpatient samples may be helpful to develop a more comprehensive understanding of the construct.

## Conclusion

This study’s findings linked parent trust deficits to BPD pathology. These results coincide with the hypothesis that deficits in epistemic trust may be a signal, and possible source, of emerging symptoms of BPD. Reduced parent trust was correlated with various self-report, parent-report, and clinician ratings of BPD after controlling for known covariates of BPD in this adolescent inpatient sample. These results are significant given that few empirical studies exist to date evaluating the impacts of deficits in epistemic trust. A key limitation of the study was the use of the IPPA trust scales, which only approximated the epistemic trust construct. Future research should address the need for a validated measure of epistemic trust in order to explore the relationship with BPD with greater precision.

## Data Availability

The datasets used and/or analyzed during the current study are available from the corresponding author on reasonable request.

## Abbreviations

BPD: Borderline Personality Disorder
BPFS-C: Borderline Personality Features Scale, Child Report
BPFS-P: Borderline Personality Features Scale, Parent Report
CBCL: Child Behavior Checklist
CIBPD: Child Interview for DSM-IV Borderline Personality Disorder
DSM-IV: Diagnostic and statistical manual of mental disorders, fourth edition
IPPA: Inventory of Parent and Peer Attachment
IPPA-M: Inventory of Parent and Peer Attachment, Mother Trust Subscale
IPPA-P: Inventory of Parent and Peer Attachment, Father Trust Subscale

## References

[1] Biskin RS (2015). The lifetime course of borderline personality disorder. Can J Psychiatr.

[2] Sharp C, Wright AGC, Fowler JC, Frueh BC, Allen JG, Oldham J (2015). The structure of personality pathology: both general (‘g’) and specific (‘s’) factors?. J Abnorm Psychol.

[3] Chanen A, Jovev M, McCutcheon L, Jackson H, McGorry P (2008). Borderline personality disorder in young people and the prospects for prevention and early intervention. Curr Psychiatr Rev.

[4] Sharp C, Fonagy P (2015). Practitioner review: borderline personality disorder in adolescence - recent conceptualization, intervention, and implications for clinical practice. J Child Psychol Psychiatry.

[5] Chanen AM, Kaess M (2012). Developmental pathways to borderline personality disorder. Curr Psychiatry Rep.

[6] Shiner RL (2009). The development of personality disorders: perspectives from normal personality development in childhood and adolescence. Dev Psychopathol.

[7] Ryle A, Kerr IB (2002). Introducing cognitive analytic therapy: principles and practice.

[8] Chanen AM, McCutcheon L (2013). Prevention and early intervention for borderline personality disorder: current status and recent evidence. Br J Psychiatry.

[9] Bateman A, Fonagy P (2009). Randomized controlled trial of outpatient mentalization-based treatment versus structured clinical management for borderline personality disorder. Am J Psychiatry.

[10] Rossouw TI, Fonagy P (2012). Mentalization-based treatment for self-harm in adolescents: a randomized controlled trial. J Am Acad Child Adolesc Psychiatry.

[11] Linehan MM (1993). Cognitive-behavioral treatment of borderline personality disorder.

[12] Miller AL, Carnesale MT, Courtney EA, Sharp C, Tackett JL (2014). Dialectical behavior therapy. Handbook of borderline personality disorder in children in adolescents.

[13] Clarkin JF, Foelsch PA, Levy KN, Hull JW, Delaney JC, Kernberg OF (2001). The development of a psychodynamic treatment for patients with borderline personality disorder: a preliminary study of behavioral change. J Personal Disord.

[14] Nomandin L, Ensink K, Yeomans F, Kernberg O, Sharp C, Tackett J (2014). Transference-focused psychotherapy for personality disorders in adolescence. Handbook of borderline personality disorder in children in adolescents.

[15] Schuppert HM, Giesen-Bloo J, van Gemert TG, Wiersema HM, Minderaa RB, Emmelkamp PMG (2009). Effectiveness of an emotion regulation group training for adolescents-a randomized controlled pilot study. Clin Psychol Psychother.

[16] Chanen A, Sharp C, Hoffman P (2017). Prevention and early intervention for borderline personality disorder: a novel public health priority. World Psychiatry.

[17] Bo S, Sharp C, Fonagy P, Kongerslev M (2017). Hypermentalizing, attachment, and epistemic trust in adolescent BPD: clinical illustrations. Personal Disord Theory Res Treat.

[18] Fonagy P, Allison E (2014). The role of mentalizing and epistemic trust in the therapeutic relationship. Psychotherapy.

[19] Fonagy P, Luyten P, Bateman A (2015). Translation: Mentalizing as treatment target in borderline personality disorder. Personal Disord Theory Res Treat.

[20] Fonagy P, Luyten P, Allison E (2015). Epistemic petrification and the restoration of epistemic trust: a new conceptualization of borderline personality disorder and its psychosocial treatment. J Personal Disord.

[21] Sperber D, Clément F, Heintz C, Mascaro O, Mercier H, Origgi G (2010). Epistemic vigilance. Mind Lang.

[22] Bateman A, Fonagy P (2012). Handbook of Mentalizing in mental health practice.

[23] Fonagy P, Gergely G, Jurist E, Target M (2002). Affect regulation, mentalization, and the development of the self.

[24] Csibra G, Gergely G (2009). Natural pedagogy. Trends Cogn Sci.

[25] Allen JG (2012). Mentalizing in the development and treatment of attachment trauma.

[26] Ensink K, Normandin L, Target M, Fonagy P, Sabourin S, Berthelot N (2015). Mentalization in children and mothers in the context of trauma: an initial study of the validity of the child reflective functioning scale. Br J Dev Psychol.

[27] Gilbert DT, Krull DS, Malone PS (1990). Unbelieving the unbelievable: some problems in the rejection of false information. J Pers Soc Psychol.

[28] Gilbert DT, Tafarodi RW, Malone PS (1993). You can’t not believe everything you read. J Pers Soc Psychol.

[29] Hasson U, Simmons JP, Todorov A (2005). Believe it or not: on the possibility of suspending belief. Psychol Sci.

[30] Corriveau KH, Harris PL, Meins E, Fernyhough C, Arnott B, Elliott L (2009). Young children’s trust in their mother’s claims: longitudinal links with attachment security in infancy. Child Dev.

[31] Miano A, Fertuck EA, Arntz A, Stanley B (2013). Rejection sensitivity is a mediator between borderline personality disorder features and facial trust appraisal. J Personal Disord.

[32] Unoka Z, Seres I, Áspán N, Bódi N, Kéri S (2009). Trust game reveals restricted interpersonal transactions in patients with borderline personality disorder. J Personal Disord.

[33] Liebke L, Koppe G, Bungert M, Thome J, Hauschild S, Defiebre N (2018). Difficulties with being socially accepted: an experimental study in borderline personality disorder. J Abnorm Psychol.

[34] King-Casas B, Sharp C, Lomax-Bream L, Lohrenz T, Fonagy P, Montague PR (2008). The rupture and repair of cooperation in borderline personality disorder. Science.

[35] Seres I, Unoka Z, Kéri S (2009). The broken trust and cooperation in borderline personality disorder. Neuroreport.

[36] Crone EA (2017). The adolescent brain: changes in learning, Descision-making, and social relations.

[37] Dahl RE (2004). Adolescent brain development: a period of vulnerabilities and opportunities. Ann N Y Acad Sci.

[38] van den Bos W, Westenberg M, van Dijk E, Crone EA (2010). Development of trust and reciprocity in adolescence. Cogn Dev.

[39] van den Bos W, van Dijk E, Crone EA (2012). Learning whom to trust in repeated social interactions: a developmental perspective. Group Process Intergroup Relat.

[40] Casey BJ, Jones RM, Hare TA (2008). The adolescent brain. Ann N Y Acad Sci.

[41] van den Bos W, van Dijk E, Westenberg M, Rombouts SARB, Crone EA (2011). Changing brains, changing perspectives. Psychol Sci.

[42] Armsden GC, Greenberg MT (1987). The inventory of parent and peer attachment: individual differences and their relationship to psychological well-being in adolescence. J Youth Adolesc.

[43] Crick NR, Murray-Close D, Woods K (2005). Borderline personality features in childhood: a short-term longitudinal study. Dev Psychopathol.

[44] Morey LC (1997). Personality assessment inventory: professional manual.

[45] Haltigan JD, Vaillancourt T (2016). The borderline personality features scale for children (BPFS-C): factor structure and measurement invariance across time and sex in a community-based sample. J Psychopathol Behav Assess.

[46] Sharp C, Mosko O, Chang B, Ha C (2010). The cross-informant concordance and concurrent validity of the borderline personality features scale for children in a community sample of boys. Clin Child Psychol Psychiatry.

[47] Chang B, Sharp C, Ha C (2011). The criterion validity of the borderline personality features scale for children in an adolescent inpatient setting. J Personal Disord.

[48] Zanarini MC (2003). Childhood interview for DSM-IV borderline personality disorder (CI-BPD).

[49] Sharp C, Ha C, Michonski J, Venta A, Carbone C (2012). Borderline personality disorder in adolescents: evidence in support of the childhood interview for DSM-IV borderline personality disorder in a sample of adolescent inpatients. Compr Psychiatry.

[50] Achenbach TM (1991). Integrative guide for the 1991 CBCL/4–18, YSR, and TRF profiles.

[51] Aggen SH, Neale MC, Røysamb E, Reichborn-Kjennerud T, Kendler KS (2009). A psychometric evaluation of the DSM-IV borderline personality disorder criteria: age and sex moderation of criterion functioning. Psychol Med.

[52] Frías Á, Palma C, Solves L, Martínez B, Salvador A (2017). Differential symptomatology and functioning in borderline personality disorder across age groups. Psychiatry Res.

[53] Ha C, Balderas JC, Zanarini MC, Oldham J, Sharp C (2014). Psychiatric comorbidity in hospitalized adolescents with borderline personality disorder. J Clin Psychiatry.

[54] Paris J (2004). Gender differences in personality traits and disorders. Curr Psychiatry Rep.

[55] Franzen N, Hagenhoff M, Baer N, Schmidt A, Mier D, Sammer G (2011). Superior “theory of mind” in borderline personality disorder: an analysis of interaction behavior in a virtual trust game. Psychiatry Res.

[56] Baldwin MW, Keelan JPR, Fehr B, Enns V, Koh-Rangarajoo E (1996). Social-cognitive conceptualization of attachment working models: availability and accessibility effects. J Pers Soc Psychol.

[57] Pierce T, Lydon JE (2001). Global and specific relational models in the experience of social interactions. J Pers Soc Psychol.

[58] Fonagy P, Luyten P (2009). A developmental, mentalization-based approach to the understanding and treatment of borderline personality disorder. Dev Psychopathol.

[59] Rotenberg KJ, Fox C, Green S, Ruderman L, Slater K, Stevens K (2005). Construction and validation of a children’s interpersonal trust belief scale. Br J Dev Psychol.

